# Prevention and Health Benefits of Prebiotics, Probiotics and Postbiotics in Acute Lymphoblastic Leukemia

**DOI:** 10.3390/microorganisms11071775

**Published:** 2023-07-08

**Authors:** Adrian Martyniak, Zuzanna Zakrzewska, Magdalena Schab, Aleksandra Zawartka, Andrzej Wędrychowicz, Szymon Skoczeń, Przemysław J. Tomasik

**Affiliations:** 1 Department of Clinical Biochemistry, Pediatric Institute, Faculty of Medicine, Jagiellonian University Medical College, 30-663 Krakow, Poland; adrian.martyniak@uj.edu.pl; 2 Department of Pediatric Oncology and Hematology, Pediatric Institute, Faculty of Medicine, Jagiellonian University Medical College, 30-663 Krakow, Poland; zuzanna.zakrzewska93@gmail.com (Z.Z.); magdalenaschab95@gmail.com (M.S.); szymon.skoczen@uj.edu.pl (S.S.); 3 Department of Paediatrics, Gastroenterology and Nutrition, Pediatric Institute, Faculty of Medicine, Jagiellonian University Medical College, 30-663 Krakow, Poland; aleksandra.zawartka94@gmail.com (A.Z.); andrzej.wedrychowicz@uj.edu.pl (A.W.)

**Keywords:** microbiota, gastrointestinal tract, inulin, pectin, anticancer, lactobacillus, fiber, folic acid, SCFA, tryptophan

## Abstract

Acute lymphoblastic leukemia (ALL) is the most common type of leukemia in children, comprising 75–85% of cases. Aggressive treatment of leukemias includes chemotherapy and antibiotics that often disrupt the host microbiota. Additionally, the gut microbiota may play a role in the development and progression of acute leukemia. Prebiotics, probiotics, and postbiotics are considered beneficial to health. The role of prebiotics in the treatment and development of leukemia is not well understood, but inulin can be potentially used in the treatment of leukemia. Some probiotic bacteria such as *Lactobacillus* shows anticancer activity in in vitro studies. Additionally, *Bifidobacterium* spp., as a consequence of the inhibition of growth factor signaling and mitochondrial-mediated apoptosis, decrease the proliferation of cancer cells. Many bacterial metabolites have promising anticancer potential. The available research results are promising. However, more research is needed in humans, especially in the child population, to fully understand the relationship between the gut microbiota and acute leukemia.

## 1. Introduction

The most common type of leukemia in children is acute lymphoblastic leukemia (ALL) comprising 75–85% of cases, with the highest incidence between 2 and 5 years of age, although it can occur at any age. In most cases of childhood, ALL (about 85%) are of B lineage. The current classification of B-line leukemia is based on seven specific genetic aberrations. Typical B-ALL aberrations include: t(12;21) [ETV6–RUNX1] t(1;19) [TCF3–PBX1], t(9;11) [BCR–ABL1]. Determining the type of mutation is very important because it has a prognostic value and determines the method of treatment. The basis for the development of leukemia is the formation of fusion genes such as ABL, ETV6, or PAX5. In addition, there is a loss of the tumor suppressor gene CDKN2A. The second type of leukemia is a T-cell line. It accounts for 10–15% of the leukemia cases in children. T-lineage leukemias are characterized by a worse prognosis and the need for more aggressive treatment with high doses of methotrexate, dexamethasone, or asparaginase. Genetic lesions in T–ALL are diverse and complex. Chromosomal aberrations are present in 50% of patients with T–ALL. Unfortunately, their prognostic value is not well defined and they are not used for risk stratification. Based on the gene expression, four major subtypes of T–ALL have been identified (TLX1, LYL1, TAL/LMO2, and TLX3). The basis of all leukemia subtypes is the formation of immature lymphoid cells, known as blasts, which are unable to differentiate into functional lymphocytes [1]. They accumulate in the bone marrow and blood and infiltrate other organs, damaging them and leading to systemic disease. The treatment of acute leukemia typically involves chemotherapy, radiation therapy, and/or stem cell transplantation. Advances in treatment strategies have led to high cure rates and currently, 5-year overall survival (OS) rates exceed 90% [2]. The method of choice in ALL treatment is chemotherapy, typically divided into three phases—induction, consolidation, and maintenance phase [3].

The gut microbiota is the collection of microorganisms that live in the gastrointestinal tract. In a state of health and normal circumstances, the microbiota of the digestive tract is composed of several strains of bacteria such as *Bacterioides*, *Fusobacteria*, *Firmicutes*, *Proteobacteria*, *Actinobacteria*, and viruses, fungi, and protozoa [4]. Many recent metagenomics studies have showed that each person’s intestinal microbiota consists of a unique collection of microorganisms that constantly interact with each other and with the host. The specific composition of individual microbiota varies extensively and depends on many factors, such as genetic variation age, diet, lifestyle, diseases, and medications [5]. Alterations in the relatively stable microbiota are called dysbiosis, which according to recent studies has a tremendous impact on human health [6]. The gut microbiota may play a role in the development and progression of acute leukemia. Changes in the gut microbiota could be associated with the response to treatment. Some studies have been undertaken in animal models—germ-free mice had several unfavorable symptoms of immune problems, i.e., defects in lymphoid tissue function, immunoglobulin A deficiency, imbalance in the T cell ratio, and disturbances in the population of hematopoietic cells [7]. However, more research is needed in humans, especially in the child population, to fully understand the relationship between the gut microbiota and acute leukemia [8]. These studies should not be limited to the analysis of general microbiota composition but also should be holistically focused on probiotics, prebiotics, and postbiotics.

### 1.1. Prebiotics

The current definition provided by the International Scientific Association for Probiotics and Prebiotics (ISAPP) indicates that a prebiotic is “a substrate that is selectively used by host microorganisms that confer a health benefit” [9]. The main criteria to classify a substance as a prebiotic include resistance to digestion and absorption in the gastrointestinal tract, lowering the pH of the intestinal content, the possibility of fermentation by intestinal bacteria and stimulating their growth, as well as a positive effect on the health of the host [10]. The classical prebiotics are galactooligosaccharides (GOS), fructooligosaccharides (FOS), human milk oligosaccharides (HMO), xylooligosaccharide (XOS), mannanoligosaccharide (MOS), and inulin. Polyphenols and polyunsaturated fatty acids (PUFA) have been the “candidate prebiotics” [9]. The prebiotics are responsible for the gut microbiota composition and therefore potentially influence, among other things, the development of acute leukemia [11].

### 1.2. Probiotics

Probiotics are live microorganisms that, when consumed in adequate amounts, can have health benefits [12]. Probiotics can produce antimicrobial substances, modulate immune system response, compete with pathogenic bacteria for adhesion to the epithelium, increase mucosal IgA production, and inhibit toxin production [13,14]. Some probiotics have also anti-inflammatory properties [15]. The effect is largely strain-dependent. Probiotics are introduced to the body along with food through fermented products such as yogurt, kefir, pickles, and kimchi, but also they are widely used in food and pharmaceutical supplements [14,16]. Probiotics may be beneficial in managing the side effects of chemotherapy and radiation therapy in acute leukemia, particularly in reducing the risk of infections and promoting gut recovery after iatrogenic damage and proper functioning.

### 1.3. Postbiotics

In 2021, a new definition of postbiotic was introduced by the International Scientific Association of Probiotics and Prebiotics (ISAPP). According to this definition, postbiotics are “a preparation of inanimate microorganisms and/or their components that confers a health benefit on the host” [17]. However, not all researchers agree with this definition, the metabolites and signaling molecules of probiotics are also commonly included as postbiotics. The properties and functions of postbiotics are determined by their chemical structure. Postbiotics are sometimes called metabiotics. The metabiotics can optimize host-specific physiological functions, and modulate metabolism, and/or behavior reactions [18]. Postbiotics are widespread in all naturally fermented foods. Numerous postbiotics can be found in kefir and yogurt, kimchi and sauerkraut, tempeh, and pickles. Postbiotics included vitamins, organic acids, short-chain fatty acids (SCFAs), and amino acids, such as tryptophan (Trp). Postbiotic acts directly or indirectly on the host [19]. The direct action is based on the interaction of the postbiotic with host cells. Indirect benefits include alterations in the environment of the gastrointestinal tract, such as acidification [20,21]. 

### 1.4. Basic Mechanisms of Anticancer Properties of Prebiotics, Probiotics, and Postbiotics

**Figure 1:**
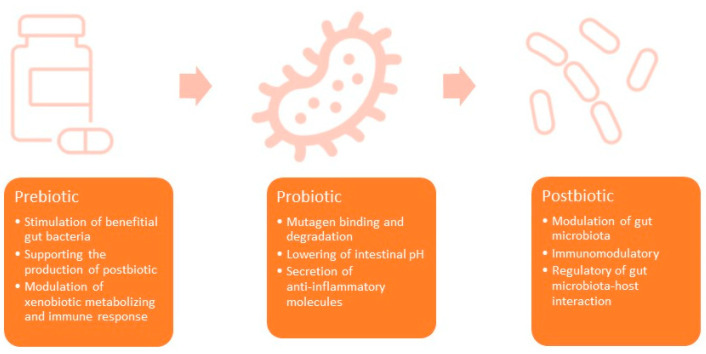
Basic anticancer properties of prebiotics, probiotics, and postbiotics.

The range of health benefits of using pre-, pro-, and postbiotics is very wide. In this article, it was decided to focus on those most useful in the treatment and prevention of ALL. Some of these properties are described in more detail below. When analyzing the potential benefits of using pre-, pro-, and postbiotics, it should be remembered that they are a group of factors with very high variability. This is especially true of the flora that colonizes the digestive tract. There are a lot of products with variable composition and documented effectiveness in use. Nevertheless, based on observations and scientific research, common mechanisms of action of pre-, pro-, and postbiotics can be found. The anti-cancer effect of prebiotics is based on (1) the stimulation of beneficial indigenous gut bacteria, (2) supporting the production of postbiotics, (3) modulation of xenobiotic metabolizing enzymes, and (4) modulation of the immune response. The general anticancer properties of probiotics include (1) mutagen binding and degradation, (2) lowering of intestinal pH, and (3) secretion of anti-inflammatory molecules [22,23]. Positive effects of using postbiotics include (1) the modulation of gut microbia, (2) immunomodulatory effects, (3) the regulation of gut microbiota–host interaction [24]. These properties are shown in Figure 1.

This paper is a summary of previous research on the impact of prebiotics, probiotics, and postbiotics on the treatment and pathogenesis of leukemia. This review focuses mainly on acute lymphoblastic leukemia since it is the most common cancer in the pediatric population in the world, and this topic has been researched primarily by scientists.

## 2. Prebiotics in ALL

The role of prebiotics in the treatment and development of leukemia is not well understood. Most research was studied in vitro or animal models. The prebiotic with potential use in the treatment of leukemia is inulin, which belongs to fructans [10]. Inulin is naturally found in bananas, onions, garlic, asparagus, artichokes, wheat, chicory, and mushrooms [25]. Schoener et al. showed that inulin in combination with doxorubicin increases the cytotoxic properties, which potentially allows for the reduction in the dose of cytostatics necessary to obtain the same therapeutic effect [26]. Bindels et al. reported that inulin supplementation in mice with leukemia increased the gut level of propionate and butyrate and reduced cancer metastases to the liver [27]. Moreover, it has been shown that inulin supplementation improves the consistency of stools in patients after radiation therapy [28]. 

Pectin is a water-soluble fiber fraction and one of the best-known plant cell wall polysaccharides [29]. Pectins have gel formation properties [30]. In food, it is found in fruits such as citrus, plums, apples, and pears, as well as in vegetables and legumes [31]. Mao et al. showed that in rats administered methotrexate, pectin supplementation significantly reduced intestinal damage, improved its integrity, reduced bacterial translocation, and reduced body weight loss [32]. A study by Bindels et al. has shown that supplementation of pectic oligosaccharides (POS) in mice with leukemia reduced anorexia associated with tumor progression, increased acetate in the cecal content, and reduced adipose tissue loss [27].

B-glucans are another type of water-soluble fiber. In food, it is found primarily in oats and barley. Cell walls of algae, bacteria, fungi, and yeast can also be their source [33]. In a study by Mao et al., it was shown that in rats administered methotrexate and oat base, the level of plasma endotoxin was reduced, body weight loss and intestinal permeability decreased, and bowel mucosal mass increased [32]. 

Lactulose is a synthetic disaccharide soluble in water, made of galactose and fructose molecules [34]. Colonic bacteria metabolize lactulose to volatile fatty acids, hydrogen, and methane, also increasing osmolarity and diminishing pH [35]. In children with ALL administration of lactulose is common in case of constipation, which is one of the most common gastrointestinal problems in this group [34]. 

## 3. Probiotics in ALL

According to research studies, ALL therapy changes the gut microbiota composition which gives rise to chronic conditions diagnosed in ALL survivals [36]. The microbiota of the gastrointestinal tract can be altered by many factors (e.g., drugs, infections, environment) leading to several conditions such as obesity, metabolic syndrome, diabetes, and cardiovascular or neurological impairments. According to Bhuta et al. (summing up available studies), there is evidence of long-term change in the microbiota of ALL survivors in comparison to the siblings’ cohort [6]. Chua et al. showed that ALL survivors (5 years after the end of therapy) had reduced microbiota diversity when compared to a healthy control group. There was also a correlation between the increase in T-cell activation and chronic inflammation which can be linked to immune dysregulation [37]. 

### 3.1. Supplementation of Probiotics in ALL

Ekert et al. conducted a study in pediatric patients with leukemia, treated with antibiotics such as framycetin, colymycin, nystatin, and metronidazole in comparison to ALL patients treated with cotrimoxazole and supplemented with Lactobacilli. Both group neither presents any significant differences in infection rate nor recovery time. In the group using Lactobacilli side effects in the form of vomiting, and nausea was diminished and resulted in better acceptance of the used medication [38]. Wada et al. evaluated the effects of *Bifidobacterium* breve strain Yakult administration in ALL patients to prevent infections. The patients presented fewer fever episodes and decreased need for antibiotic use. They also had decreased levels of Enterobacteriaceae in fecal samples [39]. Reyna-Figueroa et al. studied pediatric patients with ALL after 30 days of chemotherapy. Patients were randomly divided into two groups and half of them received *Lactobacillus rhamnosus* GG probiotic during the chemotherapy. Only 30% of patients had GI symptoms in the group who received probiotics during chemotherapy and 63% of patients from the group without probiotics presented several adverse GI effects of chemotherapy—diarrhea, nausea, vomiting, and abdominal distention. The authors noted also almost two times more cases of need for antibiotics use, hospitalization need, and septicemia in patients without probiotic supplementation as compared to the group with such a supplementation [40]. 

Besides the beneficial effects of probiotic use in oncological patients, there are also data about the adverse effect of probiotic treatment, especially in immunocompromised patients. Ambesh et al. showed that oral treatment with *Lactobacillus* species in people with an altered immune system can lead to bacteremia [39,41,42].

### 3.2. Anticancer Activity of Probiotics

Up to now, there has been a lack of data about the preventive action of probiotics against ALL, but many human, animal, and epidemiological studies have shown the prevention of colon, bladder, liver, breast, and gastric cancers with lactic acid bacteria (LAB) supplementation [43,44]. Additionally, probiotic bacteria show anticancer activity in in vitro studies using cancer cell lines. Nami et al. reported the anticancer effects of the vaginal *Lactobacillus plantarum* species on human cervical, gastric, and colon cancer cell lines. At the same time, no significant cytotoxic effects on HUVEC normal cells were observed [45]. Tarrah et al. studied eight *Streptococcus thermophilus* strains isolated from dairy environments and found that two of these strains showed anticancer activity and stimulation of folate production [46]. Similar results were found in a research study by Mangrolia et al. on Staphylococcus strains [47]. *Bifidobacterium* species, by a consequence of the inhibition of growth factor signaling and mitochondrial-mediated apoptosis, decrease the proliferation of cancer cells [48]. 

### 3.3. The Effect of Anti-ALL Therapies on Microbiota 

Aggressive therapies, as used in ALL, affect host microbiota phenotypes, mostly affecting their diversity and leading to a rise in the number of unwanted microorganisms [49,50]. During the treatment, as protocols proceeded in ALL patients, a decrease in the number and diversity of intestinal microbiota was observed. The changes were most intense in the induction phase of chemotherapy [51]. What is more, chemotherapy used in ALL treatments leads to the destruction of epithelial barrier integrity, which together with immunosuppression can cause bacteria translocation into the blood. Patients with an increased amount of Enterococcaceae in their gastrointestinal system who were undergoing chemotherapy showed an increased risk of intestinal infections [52]. Such a risk requires antimicrobial therapy, which intensifies changes in the patient’s microbiota. 

During the last years, several clinical studies have been performed that focused on diversity in the microbiota of the GI tract in pediatric patients with leukemia who underwent chemotherapy. Researchers used different types of analysis (e.g., 16S rRNA sequencing, PCR, FISH) of microbiota taken from rectal swabs or samples of feces. Studies showed a relatively large difference between the amount of microbiota in patients treated for leukemia and control groups. In a clinical study performed by Huang et al., the authors found a significant decrease in probiotic *Lactobacillus* and *Bifidobacteria*, as well as *E. coli* strains, in the intestines of children with leukemia [51]. Thomas et al. analyzed stool samples by 16S ribosomal RNA gene sequencing of patients at least 1 year after the ALL therapy with healthy siblings as a control group. They found significant differences between ALL survivors’ microbiota and their healthy siblings who served as a control group, with a huge depletion of probiotic Faecalibacterium [52]. Additionally, Rajagopala et al. noted the difference in microbiota in pediatric patients with leukemia in comparison with their healthy siblings. The differences were already present at the time of diagnosis. In both groups, they recognized Bacteroides, Prevotella, and Faecalibacterium in the stool samples, but a group of ALL patients had a severe decrease in microbiota diversity in comparison to the control group at the time of diagnosis and during chemotherapy. After one year of chemotherapy, they found a tendency to stabilization in microbiota diversity, most visible in mucolytic gram-positive bacteria (*Ruminococcus gnavus*, *Ruminococcus torques*, described as next-generation probiotics) [53]. There are also studies showing changes in certain families of gut bacteria during ALL, some of which species are harmful but others are potential probiotics. Hakim et al. recently diagnosed ALL patients’ fecal samples using V1–V3 16S rRNA gene sequencing and found an increase in Clostridiaceae and Streptococcaceae along with a decrease in the level of Bacteroidetes. They also discovered that for patients undergoing chemotherapy, a dominance of Enterococcaceae in their stool predicted febrile neutropenia, but when Streptococcaceae are dominant, it predicted diarrhea [50]. Nearing et al. used V4–V5 16S rRNA gene sequencing and metagenomic shotgun sequencing, and the results showed an increase in the number of Proteobacteria along with a decrease in Bacteroidetes and *F. prausnitzii* in the gut of ALL patients with infectious complications [54]. Chua et al. studied gut microbiota in ALL children before, during, and after chemotherapy. In children with ALL before chemotherapy, they found variability of the Bacteroidetes phylum and Bacteroides genus, which during the time of chemotherapy were decreased. However, after chemotherapy patients’ gut microbiota rebuilt their diversity, the composition was very similar to the healthy condition [55]. 

The adverse effect of chemotherapy on the gut microbiota is augmented by antibiotics. Due to a higher risk of infections, antibiotics play an important role in oncological patients; they are used as a prophylaxis (e.g., use of fluoroquinolones) but also have a therapeutic role in patients with neutropenia and fever (e.g., fourth-generation cephalosporins). Neutropenic patients for whom there was a need to use ciprofloxacin showed a high rate of *E. coli* and *K. pneumoniae* resistant to this antibiotic [56,57]. 

Besides good cure rates in ALL patients who undergo chemotherapy, there is still a group of patients who may need hematopoietic stem cell transplantation (HSCT). These procedures have a visible effect on gut microbiota diversity. There is a decrease in the number of taxa such as Faecalibacterium and Ruminococcus contrary to the rise in the number of Enterococcus, Staphylococcus, and Enterobacter [58]. Some data linked complications such as graft-versus-host disease (GVHD) with gut microbiota. Simms-Waldrip et al. described a rise in the number of Enterobacteriaceae, along with a decrease in Clostridia, revealing changes in the pediatric patients who developed GVHD [59].

The classification and effects of probiotics are summarized in Table 1.

## 4. Postbiotic in ALL

Studies of postbiotics linked straightly to leukemia are limited. Ki-Bum et al. tested kimchi extract containing postbiotics from *L. plantarum* fermentation on the human leukemia cell line HL-60, and found significant inhibition of proliferation. In the researchers’ opinion, this may be connected to a high level of ornithine in the extract [43]. Similar results were published by Hur et al.—the kimchi suppressed the growth of human leukemia cells K-562 and had no toxicity effect on normal cells [60].

Bacterial metabolites such as SCFA, exopolysaccharides, and bacteriocins have promising anticancer potential. Their anticancer activity was confirmed both in vitro and in animal studies against cancer cells from various non-ALL malignancies. LAB may inhibit tumor growth through different mechanisms, including antiproliferative activity, the induction of apoptosis, cell cycle arrest, and antimutagenic, antiangiogenic, and anti-inflammatory effects [61]. One of the beneficial effects of postbiotics produced by LAB is immunomodulatory, preventing certain types of cancer [62,63]. The effects of postbiotics include various mechanisms, such as the impact on gastrointestinal microflora by acidification of the environment, modulation of host–microbiota immune response by tryptophan metabolism, antioxidant properties of folic acid, antiproliferative action, and induction of apoptosis by SCFAs [64]. Chuah et al. tested six strains of *Lactobacillus plantarum* in different human cell lines, such as leukemia and breast cancer [65]. In their study, the microbial metabolites produced by *L. plantarum* exerted selective, time- and dose-dependent cytotoxic effects on cancer cells, without causing a toxic effect on healthy cells. 

### 4.1. Short-Chain Fatty Acids

Short-chain fatty acids are one of the most important postbiotics. SCFAs are produced by the fermentation of polysaccharides such as dietary fiber, non-starch polysaccharides (NSP), and resistant starch in the human intestine. Acetic acid (AA), butyric acid (BA), and propionic acid (PA) are the three main SCFAs. They are produced by different bacteria in almost all parts of the gastrointestinal tract, mainly in the proximal part of the large intestine. The proportions of SCFAs depended on the part of the GI tract, diet, and microbiota. Nakkarach et al. incubated human leukemia cells THP-1 with individual SCFA and *E. coli* KUB-36 metabolites for 24, 48, and 72 h. In all assays, the cytotoxicity of cancer cells was comparable [66]. The action of SCFA in ALL was also confirmed in an animal model. In a study by Song et al., the level of all SCAFs in urine samples from ALL mice were significantly lower than in the control group (for AA (*p* = 0.02), FA (*p* = 0.01), PA (*p* = 0.03), and BA (*p* = 0.05)). The author suggests that the reduced levels of SCFA in urine result from diminished production of these compounds in the small intestine of ALL mice and, in consequence, lower levels in the blood [67]. Among SCFAs, butyrate has a well-documented anti-cancer activity. It acts as a histone deacetylase inhibitor (HDAI). HDAI can normalize epigenetic disbalance by affecting gene expression. This promising strategy of new treatment based on the increase in HDAI activity has been used at the II and III stage of clinical trials. Currently, such trials are being conducted for drugs supporting the treatment of acute myeloid leukemia (AML) or T-cell leukemia [68,69]. Pulliam et al. describes the impact of a high concentration of BA (>1.5 mM) on apoptosis in various human acute leukemia cells. In U937 leukemia cells, butyrate induced a twofold activation of caspase-3 and reduced cell viability by 60%. Furthermore, within 24 h, BA significantly decreased the concentration of chemokines CCL2 and CCL5 in HL-60 and U937 cells. Additionally, concentration of CCL5 in THP-1 leukemia cells was decreased. These data promote BA as a viable therapy to induce apoptosis in leukemia cells, reduce metastasis, and regulate cytokine production in cancer [70].

### 4.2. Tryptophan

Tryptophan (Trp) and its metabolites are involved in interactions between the microbiota and the host. In the intestine, Trp can be metabolized in three ways: (1) direct metabolism by the intestinal microbiota, (2) by the host cells in kynurenine pathway, and (3) enzymatic transformation to serotonin (5-HT). Many products of Trp intestinal metabolism are ligands for the aryl hydrocarbon receptor (AhR). The most important AhR ligands are indole-3-acetic acid, tryptamine, indole-3-aldehyde (I3A), indole-3-propionic acid (IPA), indole-3-acid-acetic (IAA), indole-3-acetaldehyde (IAAl), and indoleacrylic acid. AhR is a very important transcription factor involved in the metabolism of drugs, dioxins, and other xenobiotics in the cytochrome P450 complex [71,72,73]. Tryptophan catabolism in the kynurenine pathway is directly catalyzed by the rate-limiting enzymes indoleamine 2,3-dioxygenase 1 (IDO1) and tryptophan 2,3-dioxygenase [74,75]. Sun et al. investigated the expression and function of IDO1 in AML and ALL cells in mice. In the animals treated with 1-methyl tryptophan (1-MT), significantly higher IDO expression was observed in neoplasm cells compared to normal cells, the average survival time in the experimental group was also higher (*p* < 0.05). This suggests that the pathway of tryptophan metabolism is of great importance in leukemia development [76]. In another study, Chen et al. confirmed a higher expression of IDO mRNA expression in subjects with AML and ALL compared to healthy subjects (*p* < 0.001). Higher expression of IDO leads to a low-tryptophan environment near the tissue, which reduces the ability of cells to produce certain proteins [77].

### 4.3. Folic Acid (FA)

Folates (vitamin B9) are essential micronutrients that function as cofactors in one-carbon transfer reactions involved in the synthesis of nucleotides and amino acids. Folates can be delivered to the body in three ways: supplied with food, mainly vegetables; produced by the microbiota; or provided as a drug or dietary supplement. Endogenous production of FA is possible, but insufficient. For this reason, supplementation is very important. Folic acid is a vitamin produced by several intestinal microflorae. One of the sources of synthesis in the intestines is the previously described LAB; however, most LAB species are auxotrophic for FA and other vitamins and only certain species have the ability to FA synthesis. These species can be used for food preservation and enrichment in vitamins, and the modification of a host microbiota. In this way, the need for supplementation may be reduced [78].

Folic acid is widely supplemented in pregnant women, women planning pregnancies, and postpartum women who are breastfeeding. FA is also an essential component of various types of food substitutes, such as modified milk for neonatal use [79]. Many researchers claim that childhood cancers have their origins in fetal development [80]. There are several papers confirming the protective effect of the folic acid supplementation in pregnant women on postnatal childhood cancers [81]. Shaw et al. analyzed the Canadian population and found that maternal higher levels of folic acid during and before pregnancy reduce the risk of ALL, odds ratio (OR) 0.9 (CI: 0.7–1.1) [82]. A similar analysis with a similar conclusion was performed on the German population by Schüz et al. Higher levels of FA during pregnancy have a stronger protective effect against ALL in children > 5 years of age than in younger children, with an OR 0.67 (CI: 0.5–0.9) compared to 0.98 (CI: 0.76–1.25), respectively [83]. Additionally, Amigou et al. found that high maternal FA levels before and during pregnancy can reduce the risk of ALL in childhood OR 0.4 (CI: 0.3–0.6) [84]. Singer et al. examined the association between maternal intake of multivitamins complex containing FA, B12, and B6 vitamins, riboflavin, and methionine before pregnancy. Higher maternal intake of these supplements reduced the risk of ALL in their children, OR 0.91 (CI: 0.84–0.99) [85]. In 2010 Milne et al. summarized some ALL studies and concluded that folate supplementation may have a positive effect on ALL in children, but there is no strong evidence for this. The summary OR for folate supplementation was 1.06 (CI: 0.77–1.48] compared to no folate supplementation OR 1.02 (CI: 0.86–1.20). In the case of vitamins including folate, the OR was 0.83 (CI: 0.73–0.94) [86]. Additionally, a study by Ajrouche et al. showed a minimal positive effect of FA supplementation in pregnancy on ALL in children in the future. Their study examined 646 cases of ALL in children and FA supplementation by their mothers before and during pregnancy. There are observed relationships for FA supplementation initiated in the 3 months preceding pregnancy and the risk of childhood ALL, OR 0.7 (CI: 0.5–1.0) [87]. Linabery et al. in 2010 concluded that there were no associations between FA supplementation in pregnant and childhood ALL, OR 0.63 (CI: 0.34–1.18) [88]. The effects of FA are summarized in Table 2.

## 5. Conclusions

Results of the latest research studies showed that prebiotics, probiotics, and postbiotics have multiple positive effects on the microbiota of patients with ALL. Some of the studies we cite are experimental and refer to a broader group of diseases. Others have been carried out synergistically with basic therapy. Immunosuppression, antibiotics, or diet play an important role in microbiota changes. Bacteria, such as *Lactobacillus* or *Bifidobacterium*, present some anti-cancer features. This could be due to their cooperation in the apoptosis of cancer cells, protective function in the mechanism of oxidative stress, and reduced colonization of Fusobacterium, which is a microorganism with pro-cancer properties. Probiotics can diminish the side effects of the treatment used in malignancy, e.g., mucositis, radiation-induced diarrhea, and toxicity connected with immunotherapy. Postbiotics also have a positive effect on children with ALL. High levels of FA before and during pregnancy can reduce the risk of developing ALL in childhood. Despite the undoubtedly positive effects of using prebiotics, probiotics, and postbiotics, it should be remembered that they are only complementary treatments. Biotics must not be considered the only treatment or means of prevention of ALL. Biotics, like any other drugs or medical preparations, can cause side effects and pose a danger when used incorrectly.

## Figures and Tables

**Table 1:** Probiotics and their possible positive effects.

Study	Probiotic	Effect	Therapy	Study Group
**Study on human**				
Ekert et al. [38]	*Lactobacilli* spp.	Diminished vomiting and nausea	No information about dose and time	68 children with leukemia and solid tumors
Wada et al. [39]	*Bifidobacterium breve*	Prevent infections	10^9^ freeze-dried, living BBG-01, 8 weeks	42 patients with malignancies
Reyna-Figueroa et al. [40]	*Lactobacillus rhamnosus GG*	Reducing side effects of chemotherapy	5 × 10^9^ CFU twice daily, one week	60 children with acute leukemia
**Study on cell line**				
Nami et al. [45]	*Lactobacillus plantarum*	Anticancer activity—cytotoxic effect	No data about CFU, 12, 24/48 h incubation	5 human cell line MCF-7, AGS, HeLa, HT-29, and HUVEC
Tarrah et al. [46]	*Streptococcus thermophilus*	Stimulate of folate productions	10^7^ cells/mL, 24/48 h incubation	HT-29 cell line
Mangrolia et al. [47]	*Staphylococcus xylosus*	Antibacterial and anticancer activity	No data about CFU, 24/48 h incubation	MCF-7 cell line

**Table 2:** Effects of FA intake on childhood ALL.

Study	Description	Results
Shaw et al. [82]	FA supplementation before and during pregnancy	Reduced the risk of ALL in children. OR 0.9 (CI: 0.7–1.1)
Schüz et al. [83]	FA supplementation during pregnancy	Reduced risk of ALL in children > 5 years. 0.67 (CI: 0.5–0.9)
Amigou et al. [84]	FA supplementation before and during pregnancy	Reduced the risk of ALL in childhood. OR 0.4 (CI: 0.3–0.6)
Singer et al. [85]	Maternal intake of multivitamin complex	Reduced the risk of ALL in their children. OR 0.91 (CI: 0.84–0.99)
Milne et al. [86]	Summarized some FA supplementation studies	No strong evidence for positive effects. OR 1.06 (CI: 0.77–1.48)
Ajrouche et al. [87]	FA supplementation before and during pregnancy	Minimal positive effect of FA supplementation. OR 0.7 (CI: 0.5–1.0)
Linabery et al. [88]	FA supplementation during pregnancy	No associations between FA supplementation and ALL. OR 0.63 (CI: 0.34–1.18)

## Data Availability

Data available on request from the authors.
